# Effectiveness of Pharmacist-Led Brief Educational Intervention for Adherence to the Antibiotics for Lower Respiratory Tract Infections (EATSA) in Post-Conflict Rural Areas of Pakistan: Study Protocol for a Randomized Controlled Trial

**DOI:** 10.3390/antibiotics10101147

**Published:** 2021-09-23

**Authors:** Faiz Ullah Khan, Yu Fang

**Affiliations:** 1 Department of Pharmacy Administration and Clinical Pharmacy, School of Pharmacy, Xi’an Jiaotong University, Xi’an 710061, China; fkhan@bs.qau.edu.pk; 2 Center for Drug Safety and Policy Research, Xi’an Jiaotong University, Xi’an 710061, China; 3 Shaanxi Center for Health Reform and Development Research, Xi’an 710061, China; 4 Research Institute for Drug Safety and Monitoring, Institute of Pharmaceutical Science and Technology, Western China Science & Technology Innovation Harbor, Xi’an 710061, China

**Keywords:** LRTIS, educational interventions, pharmacists, antibiotic, antibiotic resistance, Pakistan

## Abstract

Globally, lower respiratory infections (LRTIs) are one of the most common infectious diseases whichaffect majority of the population and as a result of inappropriate antibiotics practices lead to antibiotic resistance (AR). An individual randomized control trial will be conducted in the post-conflict areas of Swat, Pakistan, through a random sampling method. Patients aged > 18 years will be recruited from five community pharmacies and assigned to equally sized groups to receive either pharmacist-led education interventions or usual care with no intervention. A total of 400 (control = 200, study = 200) patients will be included, with prescriptions comprised of antibiotics for LRTIs. The outcomes measured in both groups will be a combination of treatment cure rate and adherence, which will be assessed using the Morisky Medication Adherence Scale and pill count. The trial comprises pharmacist-led educational interventions to improve treatment outcomes for patients with LRTIs. This study might establish the groundwork for pharmaceutical care of LRTIs patients with antibacterial therapy and the future delivery of a care strategy for the improvement of LRTIs treatment outcomes in post-conflict, remote areas of the third world and LMICs.

**Trial registration**: Chinese Clinical Trials Registry, identifier: ChiCTR2000040453, registered on 28 November 2020.

## 1. Introduction

Lower respiratory tract infections (LRTIs) are a type of acute sickness that lasts for 21 days or less and is characterized by the presence of at least one other lower respiratory tract symptom, in addition to the main symptom of cough (dyspnea, sputum, wheeze, or chest discomfort, and pain), with no other suitable explanation (e.g., sinusitis or asthma) [1]. Globally, LRTIs are one of the leading infectious diseases, the fifth overall cause of death, and the second most common cause of disability adjusted life years (DALYs), despite their being mainly preventable causes of mortality and morbidity [2]. Over the last ten years, the epidemiology of LRTIs has changed, with a rise in patients over the age of 70 [2]. LRTIs have no specific definitions besides the epidemiological perspective. LRTIs comprise bronchitis, acute exacerbations in chronic obstructive pulmonary disease (AECOPD), chronic obstructive pulmonary disease (COPD), pneumonia, community-acquired pneumonia (CAP), bronchiolitis, and influenza [2,3,4]. The LRTIs (2015) global burden of disease study noted that LRTIs were assessed to have caused 103.0 million DALYs and 2.74 million deaths [2]. However, pneumococcal pneumonia was found to be a cause of 55.4% of deaths in all age groups [2]. Worldwide studies from different regions, including North America [5], Latin America [6], Europe [7], and the Asia Pacific region [8], indicate the noteworthy clinical and economic load of the CAP disease. Antibiotic resistance was reported among CAP, with common pathogens, predominantly Streptococcus pneumoniae [5,8]. An African study reported that the pneumococcus remained susceptible to ampicillin/amoxicillin, which can still be used to treat the illness. The 1918 influenza pandemic killed over 50 million people, and while most influenza virus infections are self-limiting, there is still a significant number of severe infections that result in deaths, as influenza plays a considerably larger role in the etiology of CAP and AECOPD [9,10]. Moreover, patients with severe COPD and prone to acute exacerbations have more experience with antibiotics, and resistance may develop [11].

Nonetheless, antibiotics are still used for the treatment of LRTIs, resulting in the development of antimicrobial resistance [12,13]. Antimicrobials were originally regarded as lifesavers, with the best cure being antibiotics. However, incorrect antibiotic usage in the treatment of respiratory diseases might result in the emergence of resistance [14,15,16]. Antibiotics were misused in clinics because numerous isolates revealed high levels of antibiotic resistance; consequently, efficient antibiotic resistance surveillance is urgently needed to limit antibiotic abuse in China and Indonesia [17,18]. Treatment failure was more common in patients with RTIs in Pakistan because treatment outcomes are linked to antibiotic resistance [19].

Educational interventions could increase appropriate antibiotic prescription, dispensing, and/or use, which are commonly employed multimodal interventions [20,21,22]. Educational interventions at the community level reported a lower rate of antibiotic prescription, and enhanced knowledge of and attitudes towards antibiotic misuse [23]. Therefore, there has been no coverage of the topic, including educational interventions on patients recruited at community pharmacies, to improve adherence to antibiotic therapy in Pakistan. Hence, one of the barriers to care is the physical distance of rural communities from urban healthcare facilities. One of our baseline studies reported that the education of rural people is key to reducing the misuse of antibiotics [24].

The post-conflict areas (Swat) of Pakistan have witnessed extensive militancy since 2007. This has continued for many years, as the majority of the population of rural areas were displaced and migrated to the safest areas of the country [25]. During 2009, the people of Swat were asked to return after the area was cleared; however, the unremitting military presence and intermittent conflicts between security agencies and militants remain a social restiveness [26]. In the post-conflict areas of Swat, misuse of antibiotics occurs at a high frequency [24]. To address this gap, brief educational interventions will be delivered to the patients with LRTIs.

Therefore, this study aims to determine the adherence to prescribed antibiotics through educational interventions in post-conflict rural areas of Pakistan.

## 2. Materials and Methods

### 2.1. Objectives and Hypothesis

The main objective of the current trial is to test the adherence to LRTI treatment in the local/rural villages of Swat, Pakistan. The secondary objective is to assess the improvements and the success rate of the therapy. First, the quantitative part will be completed; then, a qualitative interview will be conducted.

The primary hypothesis is; the intervention group will gain knowledge of, and adhere to, the prescribed therapy for LRTIs at a higher rate than the control group at 2 and 7 weeks. The secondary hypothesis is as follows.

Post-assessment will be conducted in the second week and endpoint evaluation at the end of seventh week; participants who received the intervention are expected to report positive changes in terms of their adherence to the medication and a reduction in antibiotic storage. After the seventh week, those in the intervention group are more likely to stick to their prescribed medication. Following the quantitative phase of the research, a qualitative assessment will be conducted to determine its acceptability.

### 2.2. Study Design

**Figure 1:**
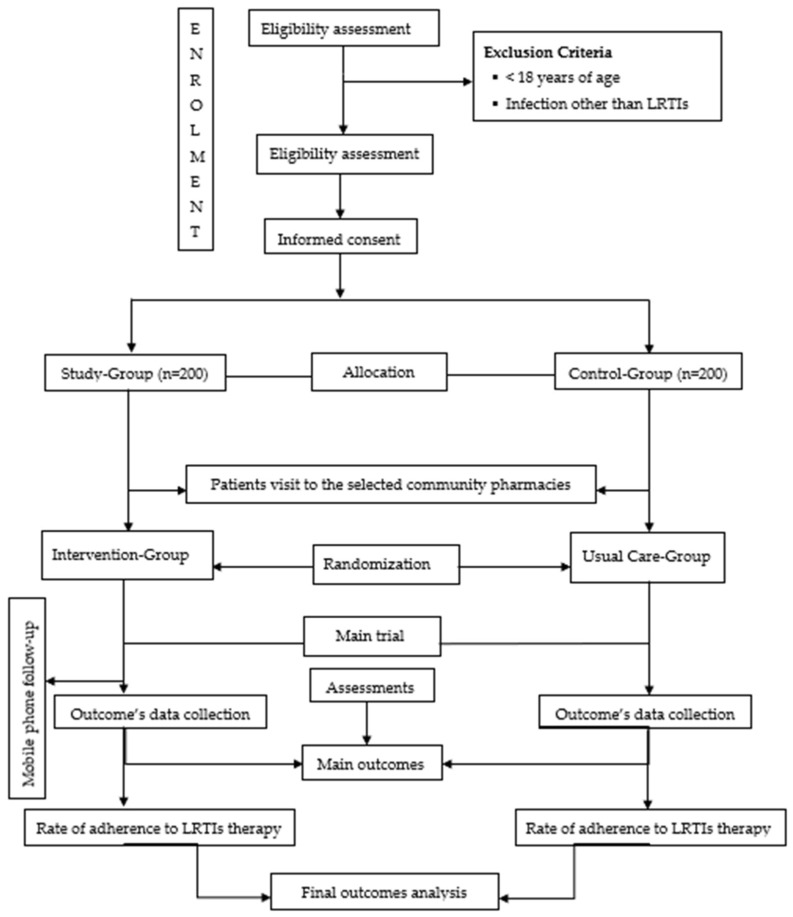
Schematic flow diagram of the trial.

This is a two-arm, single-blind, individual, randomized, controlled trial that will determine the effectiveness of pharmacist-led educational interventions for the patients who approached pharmacies with LRTI prescriptions. The present study will be conducted in a community-pharmacy setting in the rural region of Pakistan. The iRCT will be favored to reduce possible contamination between the two groups. The standard protocol items; recommendation for interventional trials (SPIRIT) used in the current trials are presented in Figure 1 and the checklist (Additional File S1).

### 2.3. Study Setting and Population

Swat, formally known as the ‘Yousufzai State of Swat’ (1849–1969), has a population of 2.3 million at present, and is one of the rural districts in the northern region of the Khyber Pakhtunkhwa, Pakistan. About 86% of the total population lives in rural areas [24,27]. Swat has witnessed extensive militancy/conflict Since 2007 [25]. In 2009, more than 2 million people in the valley became internally displaced persons (IDPs) across Pakistan during the vigorous conflict between the militants and the army [28]. In July 2009, these IDPs were asked to return, after the Government declared the area nonviolent, although the continuous military presence and the intermittent conflicts between security agencies and militants remained, creating social restlessness [26]. Infectious diseases remain high in number. The trial will be carried out in two (Khwazakhela and Janu) union councils in the Swat district, Khyber-Pakhtunkhwa Pakistan. The union council (UC) and neighborhood councils are the basic units in the new local government laws of administration in Pakistan with a population of 2000–10,000 per UC.

### 2.4. Data Collector’s Eligibility Criteria and Training

The data collector team will be comprised of healthcare workers (pharmacists, LHWs, and vaccinators). The trained pharmacists will select pharmacies (5 = control, 5 = interventional) to recruit participants, with their full consent (Additional File S2). Leaflets and materials related to the appropriate use of antibiotics will be provided to the participants at the pharmacies.

The data collector team will have the required qualifications, and the pharmacist must be registered with Pakistan Pharmacy Council (PPC) [29]. The pharmacist’s abilities are directly related to the quality of the study, which depends solely on the pharmacist’s competency. Consequently, the pharmacists involved in this study will have experience in previous patient-centered care services at community pharmacies or be engaged with population-based studies.

The pharmacists and other healthcare members must be trained for the counseling sessions with participants at community pharmacies. Effective training in communication skills will be held before the execution of the trials, and pseudo-patient training will be arranged to assess their counseling skills at community pharmacies. Finally, the principal investigator will approve the trained team for the trial.

### 2.5. Intervention Group

We have designed educational interventions and a brief, telephone-based intervention strategy will be developed for the EATSA based on WHO/CDC evidence-based guidelines for antibiotics adherence. Before its implementation, a period of formative and contextual work will be adopted, especially training in the intervention materials, to the study population in rural areas of Pakistan.

#### 2.5.1. Pharmacists Lead Educational Interventions at Community Pharmacies

Firstly, at the community pharmacy, a trained pharmacist and team will provide written information to the intervention group participants about the use of antibiotics and adherence to the prescribed antibiotics therapy for LRTIs, and discourage the storage of antibiotics at home. An educational leaflet will be designed, containing information based on ‘Get Smart: Know when Antibiotics work’ by CDC [30]. The written-material brochures adopted from the WHO/CDC awareness groups will be translated into the national and local language (Urdu/Pushto) for better understanding with the help of experts. At present, in Pakistan, patients are still deprived of proper counseling and instructions, and labels for prescribed medications are only provided as handwritten signs and descriptions. Therefore, the top section of the leaflet will have space reserved for directions on antibiotics’ use. The bottom section of the leaflet will contain information related to the importance of antibiotics’ adherence and forgetfulness, appropriate disposal, counseling on storage, the importance of not sharing the antibiotic with other people, and the risk of ABR. The leaflet’s content will be validated by two clinical pharmacists and consultant physicians specializing in infectious diseases (Figure 1). Based on the above-mentioned strategy, a full intervention will be provided to the individual LRTIs patients at time zero, and then followed to the start of the second week.

#### 2.5.2. Telephone-Based Follow-Up

After the educational interventions at the community pharmacies, a follow-up will be continued up until the 7th week. The first telephone call will be made at the start of the second week and the therapy success will measure the rate of adherence to the prescribed antibiotics. Then, a follow-up with a brief educational strategy will continue throughout the study period, and a 3rd, 4th, 5th, and 6th call will be made to enhance the participants’ knowledge related to proper antibiotics use. The 7th week of the trial will be the assessment week and will measure the overall knowledge as a primary and secondary outcome.

### 2.6. Boost Usual Care (BUC)

Participants in the intervention and control (arms) groups will continue to receive pre-planned, routine telephone calls on an individual basis and the care would be considered enhancement care. The primary care, the antibiotics therapy, will not involve any placebo, and the focus will adhere to the prescribed treatment. The usual care will consist of: (i) primary healthcare provider, pharmacists and assistants trained in interaction with and supervision of treatments with antibiotics; (ii) providing feedback results to all participants about their assessment; (iii) information will be provided about the options for appropriate care and treatment through the nearest tehsil headquarter hospital (THQ) or tertiary care facility.

Both arms are equally free to BUC through their routine health providers. Finally, on the 7th week, the strength of the services will be measured. The evaluation will be carried out and reported in a timely manner.

### 2.7. Sample Size

The previous literature will serve as a guide for calculating sample size, based on the effect of the prescribed antibiotics and pharmacist-led educational interventions on successful adherence to therapy [31,32]. A sample size of the responses will be required to give a 95% confidence interval with a 5% margin of error. Therefore, the maximum number patients from each pharmacy will be recruited until the study sample size surpasses both groups (control and interventions). Once the sample size is achieved, the principal investigator and team members will halt the patient recruitment. We will aim to achieve a 1:1 allocation ratio in the intervention or control groups, with 80%’ power and an alpha error of 5%. For the dropout participants, a 20% attrition rate will be supposed, resulting in a minimum sample size of 400 (200 in each group).

### 2.8. Recruitment of the Participants

#### Inclusion Criteria

Once their complete consent has been given, patients diagnosed with LRTIs (Table 1) will be screened for the inclusion criteria at the pharmacy. Poor adherence to recommended antibiotics for LRTIs, adult patient family members (age > 18 years), irrespective of gender, living in the UC for the last 1 year, and ability to speak and comprehend Urdu/Pushto or English are among the study’s qualifying requirements. Participants in the study group should have no extra comorbidities and have not participated in or been a part of any study/trial related to respiratory tract infections or antibiotics in the previous four months. Those who are unable to independently answer the questions owing to sight/vision, hearing, or cognitive problems, as well as those who do not meet the inclusion criteria, will be excluded (Figure 1).

### 2.9. Randomization

Using a computer-generated random digit number, a biostatistician will construct a random allocation order that is independent of/blinded to participants and trial size. For every participant that is recruited, a sample 1:1 is used to allocate them into control and interventional groups. Finally, ten pharmacists will be randomized to the intervention and control arms 1:1 and the permuted method will be used. This will be accomplished by the study teams exchanging a list of pharmacists, each of whom has a unique number assigned to them. The status of allocation will be conveyed to the research team coordinators, who will notify individual pharmacists as soon as the participants’ agreement to trial participation is obtained by research teams.

### 2.10. Concealment of Allocation

Before preceding to random allocation into either the control or intervention arm of the study, an informed consent/agreement ascertainment and eligibility will be obtained. The allocation will not be known to research assistants, participants, or trial representatives (pharmacists) until the point of randomization. Interventional households will only be known to research assistants. The participants will be introduced to the interventional arm facilitator individually, by mobile phone call. If the pharmacists and research teams are allocated to the control arm, they will continue to regularly contact the allocated UC.

### 2.11. Data Collection

At first, data from patients’ medical records reporting LRTIs (medical notes will be checked for the diagnosis) would be collected by trained pharmacists at community pharmacies, and the pharmacist team will remain blinded to the outcomes. A complete booklet of educational written information will be provided to participants in each interventional arm. The booklet will include materials that are relevant to antibiotic use, such as graphical leaflets, antibiotic dosing regimens kept on sticky notes, adherence, and prevention of antibiotic storage in the home. Following this textual counseling, oral/verbal counseling will be delivered in the patient’s usual area of counseling at a pharmacy. The patient’s information will be transferred to pre-determined data collection forms and stored securely. For the follow-up phase, patients’ valid mobile phone numbers will be collected and retained for future contact and follow-up. The trial team members will contact the participants through their mobile phones in both union councils. After one week, patients will be asked to exhibit their prescribed antibiotics, and the first therapy outcomes will be checked at the beginning of the second week. Finally, in the final session of the trial, a qualitative interview will be scheduled for the 7th week of the study. To keep data quality high, data collection should be current and dependable. As a result, at least three meetings will be held for the field assistance members (pharmacists) to keep the records up to date. The lead investigator will collect all data once a week, as in Table 2.

### 2.12. Blinding

Blinding is impossible in the present intervention, due to its educational nature. However, to reserve a certain level of blinding, to maintain and protect the bias sources, some measures have been taken. Detailed information related to blinding is shown in Table 3.

### 2.13. Participant Timeline

To control the attrition rate among patients, pharmacists will contact all intervention study participants that were enlisted via phone or text message before their scheduled follow-up session. LRTI patients who have some issues or are not prepared for a follow-up visit will be revealed, and the self-actualization survey will be completed via call if possible. The appointment can be moved to an alternate day, but the one-week treatment outcomes must be reported through mobile phone. The outcomes of the study will be based on measures of primary and secondary outcomes.

### 2.14. Outcome Measures

#### 2.14.1. Primary Outcomes

The primary outcome for individuals with LRTIs will be measured through the beliefs about medicine questionnaire (BMQ), purely to observe the participant’s beliefs about AB [33]. The BMQ was developed on a pooled basis, and its items were derived from the already published literature and interviews with ill patients [33]. Alongside the primary outcomes, the participant’s knowledge of antibiotic use, and antibiotics’ resistance will be observed through the WHO public awareness surveys questionnaire (PAS-QA). A 37-item PAS-QA will include a demographics scale (1–8) and a main QA comprising the use of antibiotics (1–8), knowledge of antibiotics (9–10 (10.1–10.6), Q12, and Q13 Likert scale (1–8, 1–6) and Q13 (antibiotics use in agriculture) [34]. Both PAS-QA and BMQ will identify the level of understanding of antibiotics among the LRTIs patients and will be measured as the primary outcome.

#### 2.14.2. Secondary Outcomes

The measure of adherence to the prescribed therapy for LRTIs will be a secondary outcome. MMAS-8 combined the adherence endpoint scale and (% age) rate of pill count; this will be used to assess the AB adherence in LRTI patients. The first seven items of the MMAS-8 adherence scale must be answered with a yes or no (dichotomous) response, while the next five questions are on a Likert scale [35,36]. The Pills Count is a simple and unconventional way to examine a patient’s adherence to a specific medication [37]. The ability to evaluate the LRTI acceptance strategy is evidenced by the inclusion of the words “non-adherent” or “adherent” on previously circulating examinations. The present examination group showed that a patient finishing the initial trial is projected to have “disciple” with an MMAS-8 ≥ 6 and a pill count rate ≥ of 80% [35].

### 2.15. Qualitative Evaluation

The trial team will conduct over 20 semi-structured interviews with a random subset of participants. The trial will include individuals with same number of dropouts and completers, and a semi-structured interviewing guide that covers a topic relevant to each respondent category. Through a mobile phone call, all interviews will be recorded (audio) through a mobile phone recorder with prior consent [38,39]. To avoid bias in the responses, the intervention teams will undertake an independent analysis of the collected qualitative interviews. Hence, the final analysis will be conducted manually, following a predetermined thematic analysis approach [38,39,40]. The schedule for the structured interviews will be finalized for each participant as per feasibility and ease, and will stop at the saturation point.

### 2.16. Trial Data Management

Data will be managed per established norms. Monitoring will be required at all stages to gather true, authentic, and verified data. Every day, the principal investigator’s field office will save the data on a paper evaluation study booklet with a participant’s given codes. The data will be checked by the lead investigator, with all highlighted queries being rectified as soon as possible, and any discrepancies being resolved by a third data entry person. Following the creation of electronic files, all data will be password-protected, and only authorized individuals will have access to the database, which will be backed up every day. The participant’s information will remain confidential to maintain quality and data secrecy. Only pharmacists will be aware of the private information in the intervention; all data forms will be kept in closed filing cabinets. The trial team will monitor the trial progress on a daily basis.

### 2.17. Statistical Analysis Methods

According to the Consolidated Standards of Reporting Trials (CONSORT) for RCTs, all the findings will be reported as per the given guidelines [41]. Data will be analyzed through IBM-SPSS-Statistics (version 21) and STATA (version 11). The basis of primary analysis is to educate the population with LRTIs, and the secondary analysis will be based on the adherence rate and behavior of the population. For the primary endpoint analysis, a linear mixed model will be employed. The mixed model will include the visits, interactions with the treatment group, and baseline measurement of BMQ and PAS-QA as covariates, and subjects as a random effect. The mean differences (MD) between the two arms at each visit, with a confidence interval (CI 95%), will be derived from a generalized mixed model, along with each visit from the treatment arm. Before unmasking the trial, all analyses will be defined in detail with their assigned statistical plans.

### 2.18. Data Dissemination

The information collected from patients, after statistical analysis, would be given to the allocated principal investigator in the field staff, who will serve as independent witnesses. The information from the research meetings in the study will become the property of the trial ethics committee, and they may request to discuss the individual information of the LRTIs patients in order to evaluate their therapy improvement. Authorship of the journal publication will be decided according to the guidelines described by the International Committee of Medical Journal Editors (ICMJE).

### 2.19. Adverse Event Monitoring

All the events related to adverse drug events (ADEs) occurring as a result of anti-infective therapy will be recorded by field staff. The ADRs will be observed and within 48 h, it will be decided whether the event occurred due to the antibiotics or other medicines. Trial staff will determine the appropriate action if necessary. The principal investigator will be in contact with the trial participants and ethical approval bodies.

## 3. Discussion

The authors are not aware of a theory that can be utilized to support the development of interventions aiming to enhance adherence to prescribed antibiotics and discourage the household storage of antibiotics in Pakistan. Hence, the overall goal of this study is to assess whether an intervention by authors with educational leaflets will enhance adherence and reduce the storage of unused antibiotics in households taking antibiotics. There is a possible association between adherence to prescribed antibiotic use and general beliefs about antibiotic storage. This study is based upon the existence of evidence that people use antibiotics in the wrong way. This is the most ignored area in the country at the community level. Additionally, the present study aims to contribute, on a humanitarian level, to the existing knowledge, along with the related factors regarding the inappropriate use of antibiotics.

Pakistan has a high rate of excessive use of medications compared to other low–middle-income countries (LMICs) with irrational prescribing, high amounts of injectables (antibiotics), costly drugs, inappropriate dispensing, and weak regulations for community pharmacies [42]. Policy targets are limited, and comprise a high rate of drug registration, poor enforcement of essential drug lists and management protocols, industry access to healthcare professionals, and private sector regulations [42]. These are the main issues from the higher to community levels, which lead to antibiotics users storing antibiotics for use without any consultation with a physician or regard to the storage conditions. These determinants lead to the inappropriate use of antibiotics and, ultimately, antibiotics resistance is a likely the result of this misuse. Hence, there is an utmost need to engage the community and all stakeholders, including the local people, to stop the irrational use of antibiotics [24,43,44,45,46,47,48].

In conclusion, once antimicrobial drugs were considered curative and lifesavers. At present, due to the global emergence of AMR, the value of these agents is under pressure. Many factors are leading to the storage of antibiotics at home, and this can raise rates of misuse, increase antibiotics’ resistance, increase wastage, and cause patients to fail to adhere to therapy.

## Figures and Tables

**Table 1:** Definition related to LRTIs.

Terms	Definition
AB (Acute Bronchitis)	A patient with no chronic lung disease, acute sickness, symptoms connected to or clinical evidence that accompanies cough, which may/may not be productive, with LRTI advised and no alternative explanation (i.e., asthma/sinusitis).
Suspected community-acquired pneumonia	Cough and fever for more than four days with at least one new symptom chest, or dyspnea, and no other obvious causes.
Definite community-acquired pneumonia	Same as the above, according to the outcomes of lung studies of chest radiographs, shadowing in the elderly, followed by acute clinical (unspecified) disease for no apparent reason.
Acute exacerbation of COPD	An occurrence in the course of the sickness characterized by the patient’s receding symptoms of cough, dyspnea, and sputum, as well as their day-to-day inconsistency, sufficient to allow for a change in treatment.
Acute exacerbation of bronchiectasis	A decline in the patient’s baseline dyspnea, sputum, and regular cough beyond inconsistency, sufficient to necessitate a change in care in a patient with bronchiectasis.
Influenza	Acute illness characterized by increased cough, body aches, headache, and sore throat, often accompanied by a fever.

**Table 2:** Schedule of enrolment, interventions, and assessment; standard protocols items: recommendations for intervention trials (SPIRIT).

	STUDY PERIOD								
	Enrollment	Allocation	Post Allocation						Close-Out Time
**TIME POINT**	**T1**	**W1**	**W2**	**W3**	**W4**	**W5**	**W6**	**W7**	
ENROLLMENT:									
Eligibility									
Consent									
Baseline Data									
Allocations									
**INTERVENTIONS:**									
leaflets			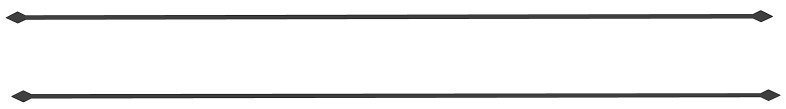						
Booklets									
WHO awareness									
Usual Care									
**ASSESSMENT:**									**Outcomes analysis**
Baseline variables	x	x	x	x	x	x	x	x	
Therapy success rate	x	x	x	x	x	x	x	x	
Primary Outcomes									
Overall Adherence Secondary Outcomes	x	x	x	x	x	x	x	x	

**Table 3:** Overall trial blinding strategies.

Stakeholders	Allocation	Pharmacist-Led Interventions	Community Pharmacists	Outcome Assessment	Data Analysis
Trial Observer (PI)	a	a	a	a	a
Trial Participants	n/a	n/a	n/a	n/a	n/a
Duty Pharmacists	n/a	a	a	n/a	n/a
Data accumulators	n/a	n/a	n/a	n/a	n/a
Data analysts	n/a	n/a	n/a	n/a	a

a: aware; n/a: not aware. PI: principal investigator.

## Data Availability

Study investigators and all the health care providers can access the patient data.

## Supplementary Materials

The following are available online at https://www.mdpi.com/article/10.3390/antibiotics10101147/s1, File S1: SPIRIT Checklist, File S2: ConsentForms (English-Urdu), File S3: Ethical Approval (ESTA).

## Trial Status and Registration

The trial status is ongoing. This study follows the SPIRIT 2013 checklist standards of reporting trials and the trial protocol is available at http://www.chictr.org.cn/ (accessed on 28 November 2020) with the assigned registered number; ChiCTR2000040453.

## Abbreviations

LRTIs: Lower respiratory tract infections, BMQ: belief about medications questionnaires, PAS-QA: public awareness surveys questionnaire, MMAS-8: Morisky Medication Adherence Scale, RCT: Randomized Controlled Trial, COPD: chronic obstructive pulmonary disease AECOPD: acute exacerbations in chronic obstructive pulmonary disease, CAP: community-acquired pneumonia, LMICs: low–middle-income countries, AMR: antimicrobial resistance, AMS: Antimicrobial stewardship.
