# Supplementary material for: Effectiveness of Pharmacist-Led Brief Educational Intervention for Adherence to the Antibiotics for Lower Respiratory Tract Infections (EATSA) in Post-Conflict Rural Areas of Pakistan: Study Protocol for a Randomized Controlled Trial
Source: Antibiotics (Basel). 2021 Sep 23;10(10):1147. doi: 10.3390/antibiotics10101147 (PMC8532944; doi:10.3390/antibiotics10101147)
Supplement: Supplementary file 1 [file antibiotics-10-01147-s001.zip › antibiotics-1350876-supplementary.pdf]

SPIRIT

STANDARD PROTOCOL ITEMS: RECOMMENDATIONS FOR INTERVENTIONAL TRIALS

SPIRIT 2013 Checklist: Recommended items to address in a clinical trial protocol and related documents*

**Section/item**	**Item No**	**Description**
**Administrative information**		
Title	1	Descriptive title identifying the study design, population, interventions, and, if applicable, trial acronym
Trial registration	2a	Trial identifier and registry name. If not yet registered, name of intended registry
	2b	All items from the World Health Organization Trial Registration Data Set
Protocol version	3	Date and version identifier
Funding	4	Sources and types of financial, material, and other support
Roles and responsibilities	5a	Names, affiliations, and roles of protocol contributors
	5b	Name and contact information for the trial sponsor
	5c	Role of study sponsor and funders, if any, in study design; collection, management, analysis, and interpretation of data; writing of the report; and the decision to submit the report for publication, including whether they will have ultimate authority over any of these activities
	5d	Composition, roles, and responsibilities of the coordinating centre, steering committee, endpoint adjudication committee, data management team, and other individuals or groups overseeing the trial, if applicable (see Item 21a for data monitoring committee)
**Introduction**		
Background and rationale	6a	Description of research question and justification for undertaking the trial, including summary of relevant studies (published and unpublished) examining benefits and harms for each intervention
	6b	Explanation for choice of comparators
Objectives	7	Specific objectives or hypotheses
Trial design	8	Description of trial design including type of trial (eg, parallel group, crossover, factorial, single group), allocation ratio, and framework (eg, superiority, equivalence, noninferiority, exploratory)

Methods: Participants, interventions, and outcomes

Study setting	9	Description of study settings (eg, community clinic, academic hospital) and list of countries where data will be collected. Reference to where list of study sites can be obtained
Eligibility criteria	10	Inclusion and exclusion criteria for participants. If applicable, eligibility criteria for study centres and individuals who will perform the interventions (eg, surgeons, psychotherapists)
Interventions	11a	Interventions for each group with sufficient detail to allow replication, including how and when they will be administered
	11b	Criteria for discontinuing or modifying allocated interventions for a given trial participant (eg, drug dose change in response to harms, participant request, or improving/worsening disease)
	11c	Strategies to improve adherence to intervention protocols, and any procedures for monitoring adherence (eg, drug tablet return, laboratory tests)
	11d	Relevant concomitant care and interventions that are permitted or prohibited during the trial
Outcomes	12	Primary, secondary, and other outcomes, including the specific measurement variable (eg, systolic blood pressure), analysis metric (eg, change from baseline, final value, time to event), method of aggregation (eg, median, proportion), and time point for each outcome. Explanation of the clinical relevance of chosen efficacy and harm outcomes is strongly recommended
Participant timeline	13	Time schedule of enrolment, interventions (including any run-ins and washouts), assessments, and visits for participants. A schematic diagram is highly recommended (see Figure)
Sample size	14	Estimated number of participants needed to achieve study objectives and how it was determined, including clinical and statistical assumptions supporting any sample size calculations
Recruitment	15	Strategies for achieving adequate participant enrolment to reach target sample size

Methods: Assignment of interventions (for controlled trials)

Allocation:

Sequence generation	16a	Method of generating the allocation sequence (eg, computer-generated random numbers), and list of any factors for stratification. To reduce predictability of a random sequence, details of any planned restriction (eg, blocking) should be provided in a separate document that is unavailable to those who enrol participants or assign interventions

Allocation concealment mechanism	16b	Mechanism of implementing the allocation sequence (eg, central telephone; sequentially numbered, opaque, sealed envelopes), describing any steps to conceal the sequence until interventions are assigned
Implementation	16c	Who will generate the allocation sequence, who will enrol participants, and who will assign participants to interventions
Blinding (masking)	17a	Who will be blinded after assignment to interventions (eg, trial participants, care providers, outcome assessors, data analysts), and how
	17b	If blinded, circumstances under which unblinding is permissible, and procedure for revealing a participant's allocated intervention during the trial
**Methods: Data collection, management, and analysis**		
Data collection methods	18a	Plans for assessment and collection of outcome, baseline, and other trial data, including any related processes to promote data quality (eg, duplicate measurements, training of assessors) and a description of study instruments (eg, questionnaires, laboratory tests) along with their reliability and validity, if known. Reference to where data collection forms can be found, if not in the protocol
	18b	Plans to promote participant retention and complete follow-up, including list of any outcome data to be collected for participants who discontinue or deviate from intervention protocols
Data management	19	Plans for data entry, coding, security, and storage, including any related processes to promote data quality (eg, double data entry; range checks for data values). Reference to where details of data management procedures can be found, if not in the protocol
Statistical methods	20a	Statistical methods for analysing primary and secondary outcomes. Reference to where other details of the statistical analysis plan can be found, if not in the protocol
	20b	Methods for any additional analyses (eg, subgroup and adjusted analyses)
	20c	Definition of analysis population relating to protocol non-adherence (eg, as randomised analysis), and any statistical methods to handle missing data (eg, multiple imputation)
**Methods: Monitoring**		
Data monitoring	21a	Composition of data monitoring committee (DMC); summary of its role and reporting structure; statement of whether it is independent from the sponsor and competing interests; and reference to where further details about its charter can be found, if not in the protocol. Alternatively, an explanation of why a DMC is not needed

	21b	Description of any interim analyses and stopping guidelines, including who will have access to these interim results and make the final decision to terminate the trial
Harms	22	Plans for collecting, assessing, reporting, and managing solicited and spontaneously reported adverse events and other unintended effects of trial interventions or trial conduct
Auditing	23	Frequency and procedures for auditing trial conduct, if any, and whether the process will be independent from investigators and the sponsor
**Ethics and dissemination**		
Research ethics approval	24	Plans for seeking research ethics committee/institutional review board (REC/IRB) approval
Protocol amendments	25	Plans for communicating important protocol modifications (eg, changes to eligibility criteria, outcomes, analyses) to relevant parties (eg, investigators, REC/IRBs, trial participants, trial registries, journals, regulators)
Consent or assent	26a	Who will obtain informed consent or assent from potential trial participants or authorised surrogates, and how (see Item 32)
	26b	Additional consent provisions for collection and use of participant data and biological specimens in ancillary studies, if applicable
Confidentiality	27	How personal information about potential and enrolled participants will be collected, shared, and maintained in order to protect confidentiality before, during, and after the trial
Declaration of interests	28	Financial and other competing interests for principal investigators for the overall trial and each study site
Access to data	29	Statement of who will have access to the final trial dataset, and disclosure of contractual agreements that limit such access for investigators
Ancillary and post-trial care	30	Provisions, if any, for ancillary and post-trial care, and for compensation to those who suffer harm from trial participation
Dissemination policy	31a	Plans for investigators and sponsor to communicate trial results to participants, healthcare professionals, the public, and other relevant groups (eg, via publication, reporting in results databases, or other data sharing arrangements), including any publication restrictions
	31b	Authorship eligibility guidelines and any intended use of professional writers
	31c	Plans, if any, for granting public access to the full protocol, participant-level dataset, and statistical code

Appendices

Informed consent materials	32	Model consent form and other related documentation given to participants and authorised surrogates
Biological specimens	33	Plans for collection, laboratory evaluation, and storage of biological specimens for genetic or molecular analysis in the current trial and for future use in ancillary studies, if applicable

*It is strongly recommended that this checklist be read in conjunction with the SPIRIT 2013 Explanation & Elaboration for important clarification on the items. Amendments to the protocol should be tracked and dated. The SPIRIT checklist is copyrighted by the SPIRIT Group under the Creative Commons "Attribution-NonCommercial-NoDerivs 3.0 Unported" license.

CONSENT FORM

(ENGLISH/URDU)

Consent Form (تحقیقی مطالعہ کے لئے رضامندی فارم)

<u>Title of Proposed Study:</u>

Effectiveness of Pharmacist led brief educational intervention for adherence to anti-infective therapy and unnecessary storage of household antibiotics in post conflicted rural areas of Pakistan.

Affiliation of researchers and contact information

**Principal Investigator** (Student)	**Research Supervisor:**
<u>Faiz Ullah Khan</u>	Prof. Dr. <u>Yu Fang</u>
Designation: Ph.D. Scholar	Designation: Professor

Affiliations (Research Collaborators): Department of Pharmacy administration and clinical Pharmacy, Xian Jiaotong University and SGTHS Swat, Khyber Pakhtunkhwa, Pakistan

Short description of the purpose and design of proposed study

Lower respiratory tract infections (LRTIs) are the leading infectious disease cause of death in the world and the fifth overall cause of death. From an epidemiological point of view, most consider pneumonia, influenza, bronchitis (including acute exacerbations in chronic obstructive pulmonary disease [AECOPD]), and bronchiolitis to be the most important LRTIs. The appropriate use of prescribed antibiotics is essential for treatment outcomes. The household storage of antibiotics leads to antibiotic resistance if leftover antibiotics stored at home for the next illness. Adherence to the prescribed antibiotics and reduction in the storage of household antibiotics are the main objectives of the present study.

دنیا میں موت کی سب سے بڑی متعدی (LRTIs) سانس کی نالی کے انفیکشن
، بیماری اور موت کی پانچواں مجموعی وجہ ہیں۔ ایک مہاماری نقطہ نظر سے
زیادہ تر نمونیا ، انفلوئنزا ، برونکائٹس)دائمی روکنےوالا پلمونری بیماری
میں شدید بڑھ جانے سمیت (اور برونکائلائٹس کو سب سے اہم [AECOPD]
تجویز کردہ اینٹی prescribed سمجھتے ہیں۔ علاج کے نتائج کے ل LRTIs
بائیوٹکس کا مناسب استعمال ضروری ہے۔ اینٹی بائیوٹکس کا گھریلو ذخیرہ
اینٹی بائیوٹک مزاحمت کا باعث بنتا ہے اگر اگلی بیماری کے لئے گھر میں بچا
ہوا اینٹی بائیوٹک محفوظ ہوجائے۔ تجویز کردہ اینٹی بائیوٹکس کی پابندی اور
گھریلو اینٹی بائیوٹکس کے ذخیرہ میں کمی موجودہ مطالعے کے بنیادی مقاصد
ہیں۔

<u>Description of the nature of risks and benefits for participants</u>

No potential risks for patients.

This study may enhance adherence to prescribed antibiotics and reduction in the storage of household antibiotics. Ultimately economic burden will decrease on patient and this study will also provide information that will be beneficial for hospital authorities, community pharmacists, and patients /community.

مریضوں کے لئے کوئی ممکنہ خطرہ نہیں ہے۔
یہ مطالعہ تجویز کردہ اینٹی بائیوٹکس کی پابندی اور گھریلو اینٹی بائیوٹکس کے
ذخیرہ میں کمی کو بڑھا سکتا ہے۔ مریض پر بالآخر معاشی بوجھ کم ہوجائے گا
، اور یہ مطالعہ ایسی معلومات بھی فراہم کرے گا جو ہسپتال کے حکام
کمیونٹی فارماسسٹ ، اور مریضوں /برادری کے لئے فائدہ مند ثابت ہوگا۔

Description of how confidentiality of the data will be assured

Any information obtained from the patient in connection with this study will remain confidential and will be disclosed only to patients and healthcare providers and the obtained information will be used only for research purpose.

اس مطالعے کے سلسلے میں مریض سے جو بھی معلومات حاصل کی گئی ہے وہ خفیہ رہے گی اور اس کا انکشاف صرف مریضوں اور صحت کی دیکھ بھال کرنے والوں کو ہوگا اور حاصل کردہ معلومات کو صرف تحقیقی مقصد کے لئے استعمال کیا جائے گا۔

Signature Statement of participant

I confirm that

1. I have read the consent form or it has been read to me and have understood to it.
2. All my questions have been answered to my satisfaction.
3. I agree to take part in the above study.

میں اس کی تصدیق کرتا ہوں

میں نے رضامندی کا فارم پڑھا ہے یا یہ میرے پاس پڑھا گیا ہے اور
اس کو سمجھ گیا ہو۔
میرے سارے سوالوں کا جواب میرے اطمینان کے مطابق دئے ہیں۔
میں مذکورہ مطالعہ میں حصہ لینے پر راضی ہوں۔

Name of Participant: نام

____

Signatures of Participant: دستخط_____________________ Date: تاریخ

Name of Researcher:

____

Signature of Researcher: _____________________ Date:

西安交通大学医学部生物医学伦理审查申请表

Xi'an Jiaotong University Health Science Center Review Application Table for Ethical Review

编号(Nọ)：2020 -

申请日期 Application date：28 January 2020

<table>
<tr><td colspan="6">项目名称：
Project Name: Effectiveness of Pharmacist led brief educational intervention for adherence to antibiotics therapy and unnecessary storage of household antibiotics (EATSA) in post conflicted rural area of Pakistan: Study protocols for Randomized Controlled Trials.</td></tr>
<tr><td colspan="4">项目负责人：
Principal investigators name： Yu Fang</td><td colspan="2">职称
Title： Professor</td></tr>
<tr><td colspan="2">电话 Tel： 18591970591</td><td colspan="4">电子信箱 Email： yufang@xjtu.edu.cn</td></tr>
<tr><td colspan="6">研究单位：
Research Units: Center for Drug Safety and Policy Research (CDSP)</td></tr>
<tr><td colspan="3">合作研究单位：
Cooperative Research Unit:</td><td colspan="3">负责人：
Principal investigators name:</td></tr>
<tr><td colspan="2">联系电话 Tel： 18591970591</td><td colspan="2">传真 Fax：</td><td colspan="2">邮编 Postcode： 710000</td></tr>
<tr><td colspan="3">研究者：
Investigator: Faiz Ullah Khan</td><td colspan="3">职称：
Title： Ph.D (Scholar)</td></tr>
<tr><td colspan="6">拟研究时间：
Research period: February 20 to December, 2020</td></tr>
<tr><td colspan="6">研究课题来源：☐政府 ☐基金会 ☐公司 ☐国际组织 ☐其他：
Research source: ☐ Government ☐ Foundation ☐ Company ☐ International organization ☐Others: Xian Jiatong University</td></tr>
<tr><td colspan="6">递交审查资料：
Submit review data: There is a need to get ethical approval for this study as we need to conduct interviews from study participants in Pakistan. There is no experimental trial on antibiotics consumers, but their interviews will be taken with their written consent. Since, it is the requirement of many journals that whenever you conduct such type of study it should be approved by the ethical committee of the university.</td></tr>
</table>

研究内容摘要

Research Abstract

Lower respiratory tract infections (LRTIs) are the leading infectious disease cause of death in the world and the fifth overall cause of death. From an epidemiological point of view, most consider pneumonia, influenza, bronchitis (including acute exacerbations in chronic obstructive pulmonary disease [AECOPD]), and bronchiolitis to be the most important LRTIs. There have been changes in the epidemiology of LRTIs in the past 10 years in that there has been a decrease in the burden in children less than 5 years of age and an increase in the burden in individuals > 70 years of age. Interestingly, pneumococcal pneumonia was found to be a cause of 55.4% of LRTI deaths among all ages.

Nowadays, the emergence and spread of resistance to antibiotics is a growing problem worldwide, which presents a significant danger to public health globally in the 21st century. The increase of antibiotics resistance will endanger their therapeutic effectiveness, increase treatment failures and, as a result, lead to longer and more severe illness episodes with higher costs and mortality rates. The irrational and overuse of antibiotics are among the key factors for the increase and spread of resistance.

The objective of the current study is to investigate the knowledge of the LRTIs participants towards antibiotics and antibiotic resistance through a mixed methodology approach from Pakistan. Educational interventions will be provided for antibiotics therapy and will fallowed.

An individual randomized trial design will be applied with a semi-structured QA will be chosen.

Different statistical tools will be used for the collected data (quantitative) and probing questions will be asked in order to get knowledge in depth (qualitative). Interviews will be transcribed verbatim and thematic analysis

A positive approach towards the anti-infective therapy in Pakistan is expected.

西安交通大学医学部医学生物科研伦理审批件

编号(No)：2020 - 1341

我部申请的项目名称：在冲突的巴基斯坦鲁拉地区，药剂师主导的简短教育干预对坚持抗生素和不必要的家庭使用抗生素（EATSA)的有效性：随机对照试验方案。

经过医学伦理委员会的审核，

符合伦理原则，同意开展。

西安交通大学医学部生物医学伦理委员会

西安交通大学医学部 生物医学伦理委员会

伦理委员会主任委员签章 陈[illegible]

2020 年1月23日

Xi'an Jiaotong University Health Science Center
Biology scientific research ethics approval

No: 2020-1341

Professor Yu Fang's application entitled "Effectiveness of Pharmacist led brief educational intervention for adherence to antibiotics therapy and unnecessary storage of household antibiotics (EATSA) in post conflicted rural area of Pakistan: Study protocols for Randomized Controlled Trials." was approved by the Medical Ethics Committee and complied with ethical principles.

Biomedical Ethics Committee of Xi'an Jiaotong University

Signature of Ethics Committee Director [illegible]

2020.11.23
